# Supplementary material for: Humans Trust Central Vision More Than Peripheral Vision Even in the Dark
Source: Curr Biol. 2019 Apr 1;29(7):1206–1210.e4. doi: 10.1016/j.cub.2019.02.023 (PMC6453110; doi:10.1016/j.cub.2019.02.023)
Supplement: Document S1. Figures S1–S3 and Table S1 [file mmc1.pdf]

**Current Biology, Volume *29***

## Supplemental Information

# Humans Trust Central Vision More Than Peripheral Vision Even in the Dark

**Alejandro H. Gloriani and Alexander C. Schütz**

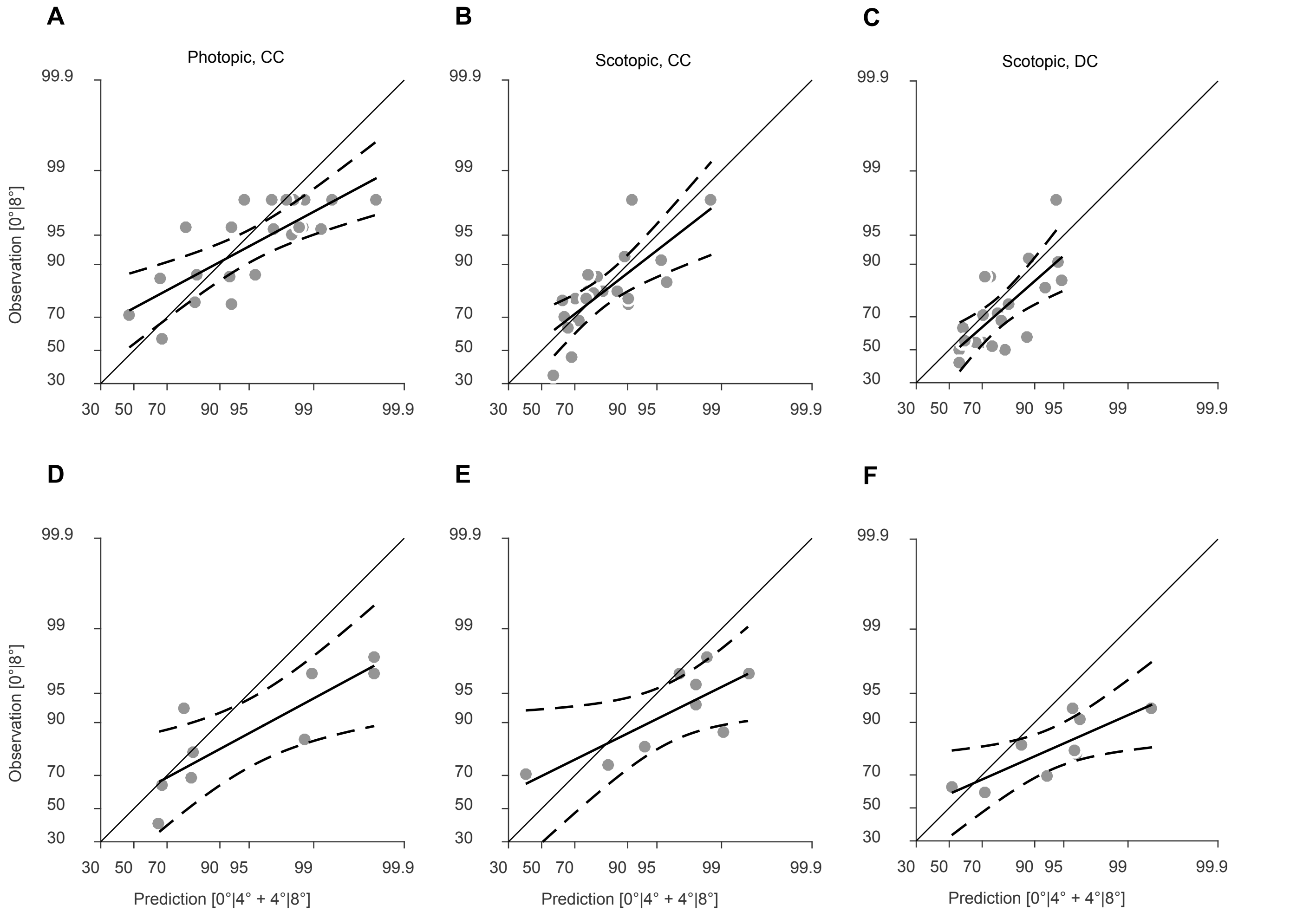


**Figure S1. Additivity of biases at different eccentricities in the comparison task of Experiment 1 and the selection task of Experiment 2 (related to Figures 2 and 3)**

The sum of the biases for 0°|4° and 4°|8° is shown on the x-axis and the bias for 0°|8° is shown on the y-axis. Before summation, a logit transformation was applied to the proportion data (Equation 1). Gray symbols indicate individual observers; the solid line indicates a linear regression and dashed lines indicate 95% confidence intervals of the regression. All regressions were significant.

(A) Experiment 1, photopic viewing, two continuous stimuli ($b$ = 0.54, $R^2$ = 0.59, $F$(1,20) = 28.21, $p$ < 0.001).

(B) Experiment 1, scotopic viewing, two continuous stimuli ($b$ = 0.77, $R^2$ = 0.56, $F$(1,20) = 24.98, $p$ < 0.001)

(C) Experiment 1, scotopic viewing, discontinuous and continuous stimuli at the lower and higher eccentricity, respectively ($b$ = 0.88, $R^2$ = 0.54, $F$(1,20) = 23.52, $p$ < 0.001). Since the DC condition at 4°|8° was not ambiguous, we used the CC condition at 4°|8° for this prediction.

(D) Experiment 2, selection task, photopic viewing, two continuous stimuli ($b$ = 0.54, $R^2$ = 0.71, $F$(1,7) = 17.02, $p$ = 0.004).

(E) Experiment 2, selection task, scotopic viewing, two continuous stimuli ($b$ = 0.49, $R^2$ = 0.60, $F$(1,7) = 10.69, $p$ = 0.014).

(F) Experiment 2, selection task, scotopic viewing, discontinuous and continuous stimuli at the lower and higher eccentricity, respectively ($b$ = 0.44, $R^2$ = 0.66, $F$(1,7) = 13.35, $p$ = 0.008). Since the DC condition at 4°|8° was not ambiguous, we used the CC condition at 4°|8° for this prediction.

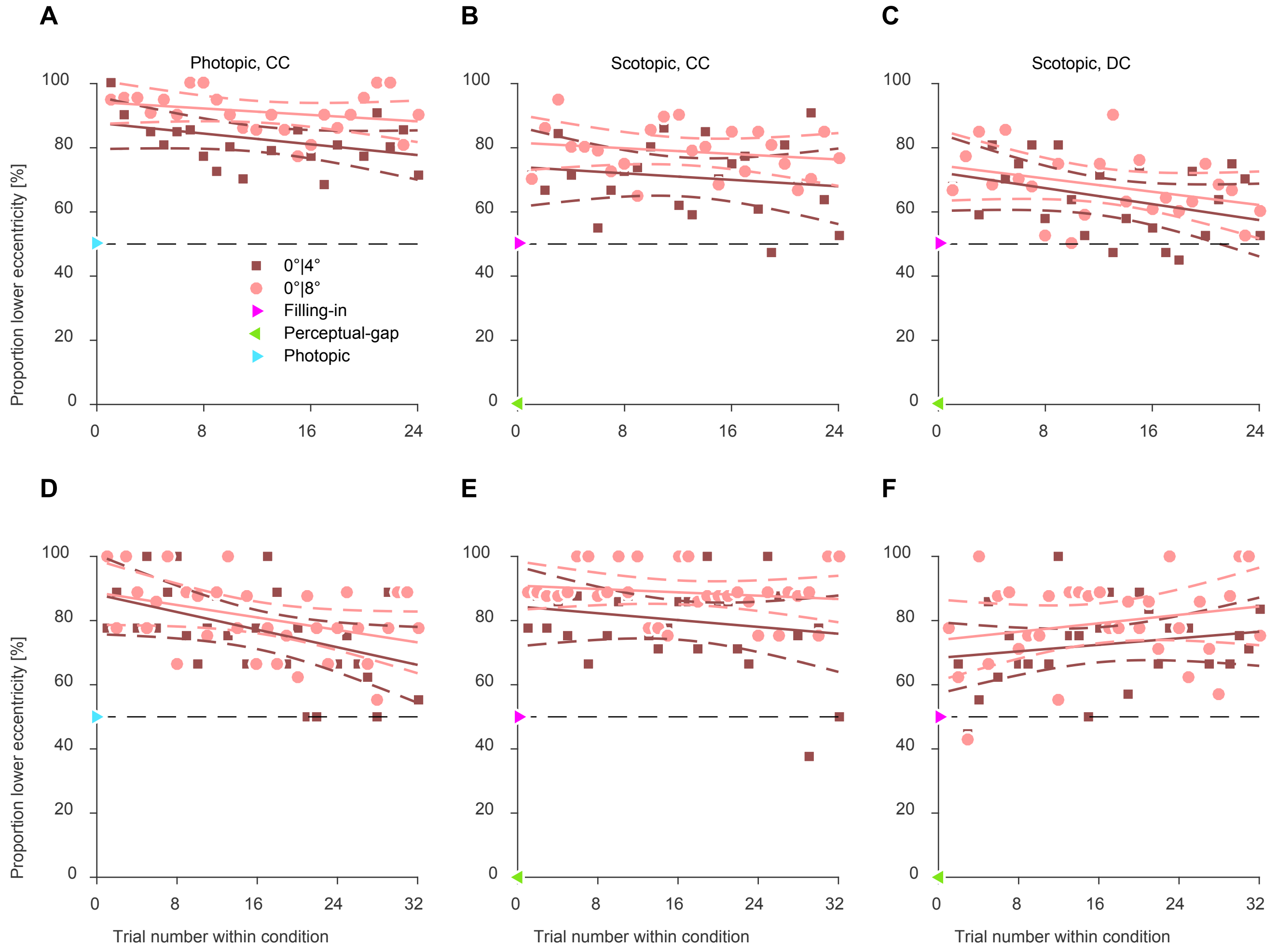


**Figure S2. Distribution of biases across trials in the comparison task of Experiment 1 and the selection task of Experiment 2 (related to Figures 2 and 3)**

Circles and squares show averages across observers for each trial within a condition. The solid line indicates a linear regression and dashed lines indicate 95% confidence intervals of the regression. 0°|4° and 0°|8° conditions are shown in dark and bright red, respectively. Triangles indicate predictions (STAR Methods) if observers trust the filled-in information at the fovea (magenta) or either do not fill-in or do not trust the filled-in information (green) or if they have veridical foveal information under photopic viewing (cyan).

(A) Experiment 1, photopic viewing, two continuous stimuli (0°|4°: $b = -0.42$, $R^2 = 0.14$, $F(1,22) = 3.66$, $p = 0.069$; 0°|8°: $b = -0.25$, $R^2 = 0.08$, $F(1,22) = 1.86$, $p = 0.186$).

(B) Experiment 1, scotopic viewing, two continuous stimuli (0°|4°: $b = -0.25$, $R^2 = 0.03$, $F(1,22) = 0.57$, $p = 0.459$; 0°|8°: $b = -0.22$, $R^2 = 0.04$, $F(1,22) = 0.87$, $p = 0.361$)

(C) Experiment 1, scotopic viewing, discontinuous and continuous stimuli at the lower and higher eccentricity, respectively (0°|4°: $b = -0.62$, $R^2 = 0.14$, $F(1,22) = 3.72$, $p = 0.067$; 0°|8°: $b = -0.52$, $R^2 = 0.12$, $F(1,22) = 3.02$, $p = 0.096$).

(D) Experiment 2, selection task, photopic viewing, two continuous stimuli (0°|4°: $b = -0.69$, $R^2 = 0.20$, $F(1,30) = 7.27$, $p = 0.011$; 0°|8°: $b = -0.49$, $R^2 = 0.15$, $F(1,30) = 5.50$, $p = 0.026$).

(E) Experiment 2, selection task, scotopic viewing, two continuous stimuli (0°|4°: $b = -0.27$, $R^2 = 0.03$, $F(1,30) = 1.08$, $p = 0.308$; 0°|8°: $b = -0.13$, $R^2 = 0.02$, $F(1,30) = 0.70$, $p = 0.411$).

(F) Experiment 2, selection task, scotopic viewing, discontinuous and continuous stimuli at the lower and higher eccentricity, respectively (0°|4°: $b = 0.25$, $R^2 = 0.04$, $F(1,30) = 1.24$, $p = 0.274$; 0°|8°: $b = 0.33$, $R^2 = 0.05$, $F(1,30) = 1.59$, $p = 0.217$).

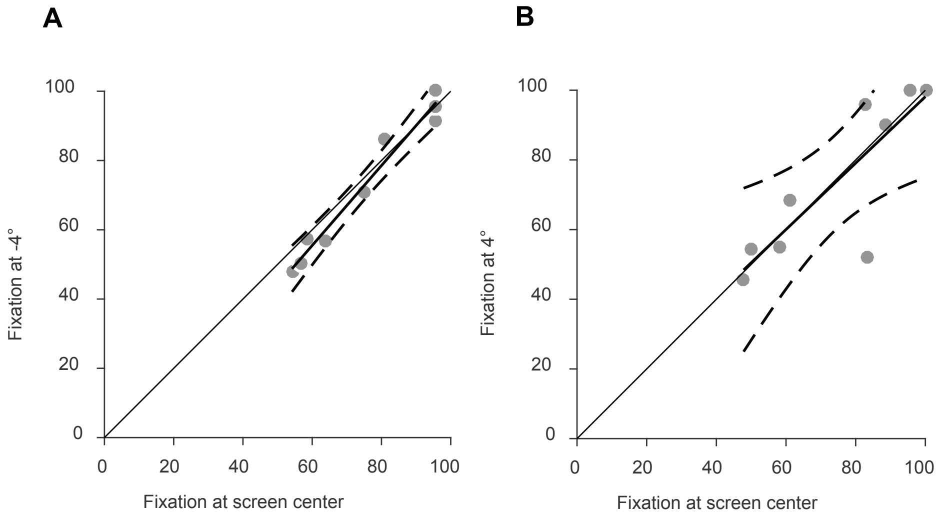


**Figure S3. Influence of retinal and screen location on perceptual biases in Experiment 3 (n=9) (related to STAR Methods)**

To study if perceptual biases depended on the location on the screen or on the retina, we varied the fixation location in three conditions (-4°, 0° and 4°) under photopic viewing. In each trial two continuous stimuli were presented at the fixation location and either 4° left or right from fixation. This way we could compare two conditions with matching stimulus locations at the screen, but one with fixation at the screen center and one with fixation 4° left or right from the screen center. For each condition we calculated the proportion of the fixated stimulus reported as more continuous. If biases depend on retinal location, there should be a positive relationship between central and eccentric fixation conditions. If biases depend on screen location, there should be a negative relationship between central and eccentric fixation conditions. Gray symbols indicate individual observers; the solid line indicates a linear regression and dashed lines indicate 95% confidence intervals of the regression. (A) Fixation at screen center vs. fixation at -4° eccentricity ($b = 1.15$, $R^2 = 0.97$, $F(1,7) = 203.68$, $p < 0.001$). (B) Fixation at screen center vs. fixation at 4° eccentricity ($b = 0.95$, $R^2 = 0.70$, $F(1,7) = 16.40$, $p = 0.005$).

| Condition | | 1 | 2 | 3 | 4 | 5 | 6 | 7 | 8 | 9 | 10 | 11 | 12 | 13 | 14 | 11 | 12 | 17 | 18 | 19 | 20 | 21 | 22 | 23 | 24 |
|---|---|---|---|---|---|---|---|---|---|---|---|---|---|---|---|---|---|---|---|---|---|---|---|---|---|
| Location | | Same eccentricity | | | | | | Different eccentricity | | | | | | | | | | | | | | | | | |
| Stimulus | | | | | | | | CD | | CD | | CD | | DC | | DC | | DC | | CC | | CC | | CC | |
| 1st | Ecc | 0 | | 4 | | 8 | | 0 | 4 | 0 | 8 | 4 | 8 | 0 | 4 | 0 | 8 | 4 | 8 | 0 | 4 | 0 | 8 | 4 | 8 |
| | Rot | 90 | 0 | 90 | 0 | 90 | 0 | 0 | 90 | 0 | 90 | 0 | 90 | 90 | 0 | 90 | 0 | 90 | 0 | 0 | 0 | 0 | 0 | 0 | 0 |
| 2nd | Ecc | 0 | | 4 | | 8 | | 4 | 0 | 8 | 0 | 8 | 4 | 4 | 0 | 8 | 0 | 8 | 4 | 4 | 0 | 8 | 0 | 8 | 4 |
| | Rot | 0 | 90 | 0 | 90 | 0 | 90 | 90 | 0 | 90 | 0 | 90 | 0 | 0 | 90 | 0 | 90 | 0 | 90 | 0 | 0 | 0 | 0 | 0 | 0 |

**Table S1. Conditions in Experiments 1 and 2 (related to Figure 1 and STAR Methods)**
Condition: The temporal order of the dark shaded conditions was reversed when averaging out sequence effects. Location: Same eccentricity conditions were only tested in Experiment 1. Stimulus: C indicates continuous, D indicates discontinuous; light shaded labels indicate ambiguous conditions (both stimuli perceived as continuous) in scotopic and photopic viewing; dark shaded labels indicate ambiguous conditions (both stimuli perceived as continuous) only in scotopic viewing. Ecc.: Eccentricity of stimulus in °. Rot.: Difference between center and surround orientation in °.
