# Supplementary material for: Humans Trust Central Vision More Than Peripheral Vision Even in the Dark
Source: Curr Biol. 2019 Apr 1;29(7):1206–1210.e4. doi: 10.1016/j.cub.2019.02.023 (PMC6453110; doi:10.1016/j.cub.2019.02.023)
Supplement: Document S2. Article plus Supplemental Information [file mmc2.pdf]

Current Biology

Report

Humans Trust Central Vision More Than Peripheral Vision Even in the Dark

Graphical Abstract

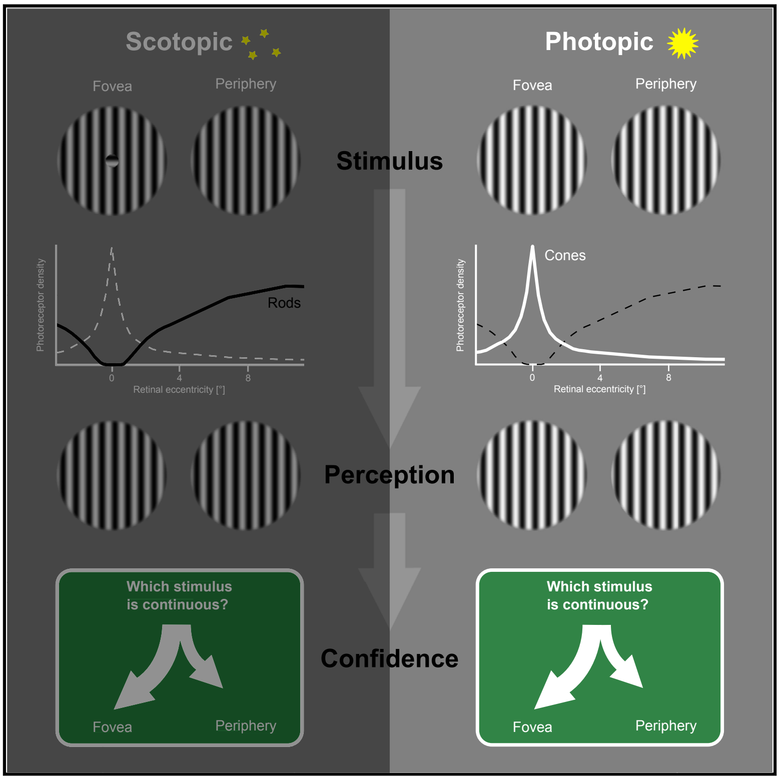

Highlights

- Veridical information from the fovea is preferred under photopic viewing
- Information missing in the scotopic foveal scotoma is filled in from the surround
- Inferred information from the fovea is preferred under scotopic viewing
- Content and properties of the foveal scotopic scotoma are hidden from awareness

Authors

Alejandro H. Gloriani, Alexander C. Schütz

Correspondence

a.schuetz@uni-marburg.de

In Brief

Gloriani and Schütz show that the absence of rod photoreceptors in central vision is compensated by filling-in of information from the surround under dark adaptation. Human observers trust this inferred information more than veridical information from peripheral vision. This preference for central vision is also present under daylight conditions.

Gloriani & Schütz, 2019, Current Biology 29, 1206–1210
April 1, 2019 © 2019 The Author(s). Published by Elsevier Ltd.
https://doi.org/10.1016/j.cub.2019.02.023

CellPress

Current Biology
Report

Humans Trust Central Vision More Than Peripheral Vision Even in the Dark

Alejandro H. Gloriani[1] and Alexander C. Schütz[1,2,*]
[1]Department of Psychology, University of Marburg, Gutenbergstr. 18, 35032 Marburg, Germany
[2]Lead Contact
*Correspondence: a.schuetz@uni-marburg.de
https://doi.org/10.1016/j.cub.2019.02.023

SUMMARY

Two types of photoreceptors in the human retina support vision across a wide range of luminances: cones are active under bright daylight illumination (photopic viewing) and rods under dim illumination at night (scotopic viewing). These photoreceptors are distributed inhomogeneously across the retina [1]: cone-receptor density peaks at the center of the visual field (i.e., the fovea) and declines toward the periphery, allowing for high-acuity vision at the fovea in daylight. Rod receptors are absent from the fovea, leading to a functional foveal scotoma in night vision. In order to make optimal perceptual decisions, the visual system requires knowledge about its own properties and the relative reliability of signals arriving from different parts of the visual field [2]. Since cone and rod signals converge on the same pathways [3], and their cortical processing is similar except for the foveal scotoma [4], it is unclear if humans can take into account the differences between scotopic and photopic vision when making perceptual decisions. Here, we show that the scotopic foveal scotoma is filled in with information from the immediate surround and that humans trust this inferred information more than veridical information from the periphery of the visual field. We observed a similar preference under daylight illumination, indicating that humans have a default preference for information from the fovea even if this information is not veridical, like in night vision. This suggests that filling-in precedes the estimation of confidence, thereby shielding awareness from the foveal scotoma with respect to its contents and its properties.

RESULTS

Experiment 1: Comparison Task

In experiment 1 (Figure 1), observers saw two stimuli consecutively and had to indicate which one appeared to them as continuous [5]. Each stimulus consisted of a striped center and surround, which could have the same (continuous) or the orthogonal (discontinuous) orientation. In different sessions, observers saw the stimuli under dark-adapted conditions, where vision is mediated by rods (scotopic viewing), or under light-adapted conditions, where vision is mediated by cones (photopic viewing). The size of the stimulus center was chosen, such that it would fall completely into the foveal scotoma, when the stimulus was presented centrally under scotopic viewing.

To measure objective discrimination performance, a continuous and a discontinuous stimulus were presented consecutively at the same location, and we calculated the proportion of correct decisions (Figure 2A). Under scotopic viewing, observers were only slightly better than chance performance of 50% at the fovea (56.6%, confidence interval $[CI]_{95\%}$ [51.9, 60.7]) but close to perfect performance at 4° (94.9%, $CI_{95\%}$ [92.3, 97.0]) and 8° (96.6%, $CI_{95\%}$ [94.6, 98.1]) eccentricity. Thus, observers could not veridically detect the discontinuous stimulus center inside their scotopic foveal scotoma either due to a perceptual gap [6, 7] or due to filling-in [8, 9] of information from the surround [10]. Under photopic viewing, observers were close to perfect performance at all eccentricities.

To measure the relative weighting of different eccentricities, we interleaved conditions with a continuous and a discontinuous stimulus at two different eccentricities (0°|4°, 0°|8° or 4°|8°) and calculated the proportion of lower-eccentricity choices (Figure 2B). We predicted that if observers experience a perceptual gap at the scotopic foveal scotoma or experience filling-in but do not trust this inferred information (perceptual-gap), they should base their decisions on peripheral information and ignore foveal information. If observers experience filling-in and trust this inferred information (filling-in), they should weight foveal and peripheral information equally (STAR Methods).

When the continuous stimulus was less eccentric, observers almost always chose this stimulus as continuous under both photopic and scotopic viewing, consistent with all predictions. When the discontinuous stimulus was less eccentric, observers almost never chose this stimulus as continuous under photopic viewing. As predicted, this was also the case for the 4°|8° condition under scotopic viewing. A foveal stimulus, however, was reported more often as continuous than stimuli at 4° (64.4%, $CI_{95\%}$ [57.3, 71.3]) or 8° (68.1%, $CI_{95\%}$ [61.4, 75.3]) under scotopic viewing, which violates the perceptual-gap prediction and exceeds even the filling-in prediction. This does not reflect a general response bias for less eccentric stimuli because half of the observers each had to report which stimulus was continuous or which was discontinuous. Instead, the observed bias suggests that the scotopic foveal scotoma was filled in and that observers trusted this inferred information even more than the veridical information from the periphery.

 © 2019 The Author(s). Published by Elsevier Ltd.
This is an open access article under the CC BY license (http://creativecommons.org/licenses/by/4.0/).

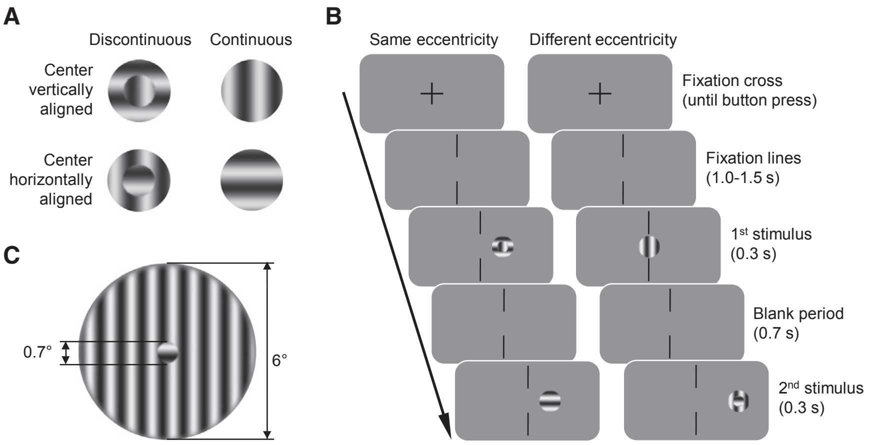

Figure 1. Stimuli and Experimental Procedure

(A) Four possible stimulus configurations with discontinuous or continuous center and surround and vertical or horizontal orientation (see also Table S1).

(B) Experimental procedure with stimuli at the same eccentricity (experiment 1) and stimuli at different eccentricities (experiments 1 and 2). In experiment 1, observers had to indicate whether the first or the second stimulus appeared continuous (or discontinuous, half of the observers each). In experiment 2, observers had to select initially if they want to judge the first or the second stimulus and indicate subsequently if the selected stimulus appeared continuous or discontinuous.

(A and B) Stimuli are not drawn to scale.

(C) Example of a discontinuous stimulus drawn to scale.

To measure perceptual biases not only under scotopic viewing, but also under photopic viewing, we interleaved ambiguous conditions with two continuous stimuli. Here, observers reported more often the less eccentric stimulus as continuous under scotopic viewing (0°|4°: 70.9%, $CI_{95\%}$ [65.9, 76.8]; 0°|8°: 78.4%, $CI_{95\%}$ [70.7, 83.5]; 4°|8°: 63.1%, $CI_{95\%}$ [59.5, 66.8]), as well as photopic viewing (0°|4°: 82.5%, $CI_{95\%}$ [77.5, 87.5]; 0°|8°: 90.9%, $CI_{95\%}$ [84.8, 94.6]; 4°|8°: 73.7%, $CI_{95\%}$ [64.9, 80.2]), which again exceeds the filling-in prediction. To summarize, observers were more likely to report the less eccentric stimulus as continuous under all ambiguous conditions irrespective of whether the ambiguity was real because two continuous stimuli were presented or whether it was inferred because a discontinuous stimulus was shown at the scotopic foveal scotoma.

In the 0°|8° condition, biases were largest and could be accounted for by a sum of the biases of 0°|4° and 4°|8° (Figure S1). Therefore, the biases peak in central vision and decline with eccentricity. A trial-by-trials analysis showed that the biases were already present in the first few trials and did not change substantially across the experiment (Figure S2), indicating that they were not acquired during the experiment. A control experiment showed that the biases depend on the stimulus location on the retina but not on the screen (Figure S3 and STAR Methods) and therefore are not caused by inhomogeneities of luminance or contrast across the screen but reflect an intrinsic property of perceptual decision making.

Experiment 2: Selection and Appearance Task

The comparison task in experiment 1 combines two aspects in one decision: observers had to decide which stimulus appeared continuous and, if both of them appeared continuous, which stimulus they felt more confident about. This makes the measurement very efficient, but it does not explicitly separate appearance (type 1 judgments) from confidence (type 2 judgments) (reviewed in [11]). To further separate these aspects, observers had to make two consecutive decisions in experiment 2: first, they had to select which of the two stimuli they want to judge (selection task, type 2 judgment), and then, they had to report if this selected stimulus was continuous or discontinuous (appearance task, type 1 judgment) [12].

In the selection task (Figure 3A), we analyzed the proportion of selecting the less eccentric stimulus. We predicted that if observers experience a perceptual gap at the scotopic foveal scotoma or experience filling-in but do not trust this inferred information (perceptual-gap), they should select peripheral stimuli and avoid foveal stimuli. If observers experience filling-in and trust this inferred information (filling-in), they should select foveal and peripheral stimuli equally often (STAR Methods).

When the continuous stimulus was less eccentric than the discontinuous stimulus, selections were quite variable between observers and not different from 50% under scotopic and photopic viewing. When the discontinuous stimulus was less eccentric than the continuous stimulus, there were also no consistent preferences in any photopic condition and in the 4°|8° condition under scotopic viewing. Since these conditions involve the selection between two different types of stimuli (continuous versus discontinuous), potential preferences for stimulus eccentricity might be obscured by large interindividual differences in preferences for stimulus type. To circumvent this issue, we calculated the proportion of selecting the less eccentric stimulus independently of whether the discontinuous or the continuous stimulus was less eccentric. This averaged out the effect of stimulus type and revealed consistent preferences for the less eccentric stimulus under photopic (0°|4°: 65.8%, $CI_{95\%}$ [58.9, 74.8]; 0°|8°: 71.8%, $CI_{95\%}$ [61.6, 81.7]; 4°|8°: 60.0%, $CI_{95\%}$ [51.8, 70.5]) and scotopic (4°|8°: 56.0%, $CI_{95\%}$ [51.1, 68.0]) viewing.

When the discontinuous stimulus was presented in the scotopic foveal scotoma (Figure 3A), observers selected it more often (0°|4°: 72.8%, $CI_{95\%}$ [58.9, 88.3]; 0°|8°: 79.3%, $CI_{95\%}$ [70.8, 86.2]). In trials with two continuous stimuli, observers also preferentially selected the less eccentric stimulus under scotopic (0°|4°: 81.0%, $CI_{95\%}$ [64.4, 90.7]; 0°|8°: 88.8%, $CI_{95\%}$ [81.4, 94.5]; 4°|8°: 78.1%, $CI_{95\%}$ [67.3, 83.8]) and photopic (0°|4°: 76.9%, $CI_{95\%}$ [68.3, 86.9]; 0°|8°: 80.8%, $CI_{95\%}$ [64.7, 90.4]; 4°|8°: 74.7%, $CI_{95\%}$ [62.6, 85.9]) viewing, violating the perceptual-gap prediction and exceeding the filling-in prediction. Like in experiment 1, the preferences in 0°|8° could be accounted for by the sum of preferences in 0°|4° and 4°|8° (Figure S1), and they were stable over the course of the experiment (Figure S2). To summarize, when observers had to choose between two stimuli for their perceptual

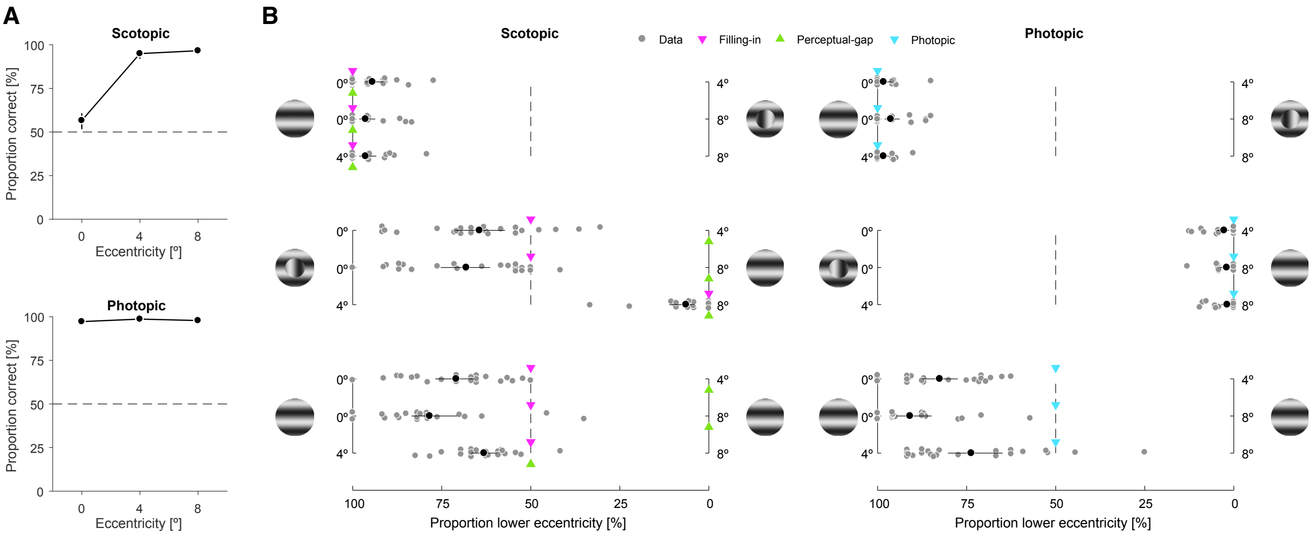

Figure 2. Results from the Comparison Task in Experiment 1 (n = 22)

(A) Proportion of correct responses with two stimuli at the same eccentricity.

(B) Proportion of less eccentric stimuli reported as continuous. Stimulus examples illustrate which stimulus was shown at the respective eccentricity, with less eccentric stimuli are shown on the left. Triangles indicate predictions (STAR Methods) if observers trust the filled-in information at the fovea (magenta) or either do not fill in or do not trust the filled-in information (green) or if they have veridical foveal information under photopic viewing (cyan).

Gray symbols indicate individual observers; black symbols the mean across observers. Error bars are bootstrapped 95% CIs. Dashed lines indicate guessing without a bias. See also Figures S1, S2, and S3.

judgment, they consistently preferred the less eccentric stimulus when the stimuli were identical or when a discontinuous stimulus was presented at the foveal scotopic scotoma. This indicates that observers were not aware of their foveal scotopic scotoma and that they trusted central information more than peripheral information under scotopic and photopic viewing.

In the subsequent appearance task (Figure 3B), observers reported all stimuli correctly, except for discontinuous stimuli presented in the scotopic foveal scotoma, which were reported as continuous in most of the cases (90.0%, $CI_{95\%}$ [81.0, 95.9]). These results of the appearance task provide further evidence for filling-in in the scotopic foveal scotoma.

DISCUSSION

We investigated perceptual decision making under scotopic and photopic viewing. In two experiments, we found that a stimulus with a discontinuity in the scotopic foveal scotoma appeared as continuous, providing evidence for perceptual filling-in of the scotoma. We also found that observers preferred information from central vision, even when it was not veridical under scotopic viewing. This general preference for central vision indicates that humans are not aware of their scotopic foveal scotoma and that it is not taken into account for perceptual decision making.

Under daylight illumination, basic perceptual measures, such as acuity [13] or contrast sensitivity [14], peak at the fovea and decline in the periphery. In addition, the periphery is more vulnerable to crowding—i.e., spatial interference between neighboring elements [15]. Preferring information from central vision might be, therefore, a sensible strategy for decision making under ambiguity in photopic vision. This interpretation is supported by other foveal biases in photopic vision: stimuli with temporal and spatial uncertainty tend to be mislocalized toward the fovea [16], foveal brightness is extrapolated into the periphery [17], peripheral appearance is influenced by predicted foveal appearance [18, 19], and transsaccadic feature integration shows some overweighting of foveal information [20, 21]. However, the observed perceptual bias is not a useful strategy for scotopic vision, where the fovea does not contribute veridical information. Nevertheless, our finding is consistent with other perceptual phenomena where vision in the light and the dark is not calibrated well: perceived speed is underestimated in the dark [22], and the perception of white seems to require signals from cones [23]. Our results are at odds with a recent comparison of photopic and scotopic visual search [24], where eye movement statistics are affected by lighting condition in a qualitatively similar way as an ideal searcher [25], which has knowledge about the scotopic foveal scotoma. These divergent findings could point toward a general dissociation that the scotopic foveal scotoma is taken into account in eye movement control, but not in perceptual decision making. Alternatively, the divergent findings might be caused by different opportunities for learning in the two experimental paradigms. In the visual search task, observers experienced with every eye movement how visual input in the fovea and the periphery relate to each other and therefore had the opportunity to acquire the appropriate weighting of foveal and peripheral information. In the perceptual decision task of the current study, observers never experienced the same stimulus in the fovea and the periphery and therefore could not acquire the appropriate weighting during the experiment.

There are at least two ways how the perceptual bias could be caused in scotopic vision: first, the brain might use a simple heuristic that information from the fovea is more reliable than from the periphery and apply this heuristic to photopic and scotopic

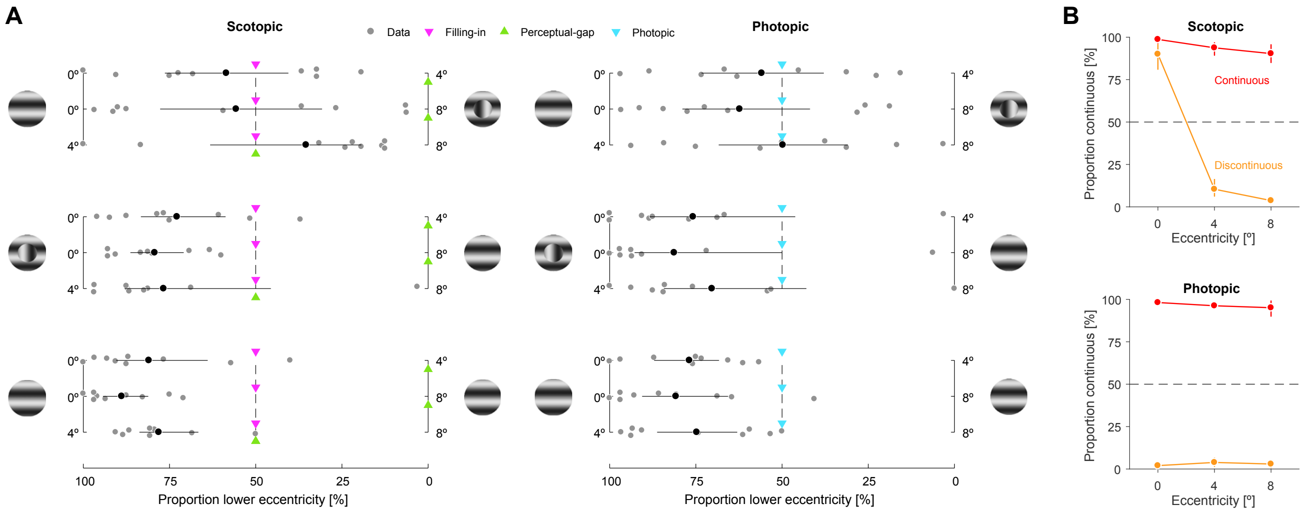

Figure 3. Results from Experiment 2 (n = 9)

(A) Selection task: proportion of less eccentric stimuli selected for report in the subsequent appearance task. Stimulus examples illustrate which stimulus was shown at the respective eccentricity; less eccentric stimuli are shown on the left. Gray symbols indicate individual observers; black symbols indicate the mean across observers. Triangles indicate predictions (STAR Methods) if observers trust the filled-in information at the fovea (magenta) or either do not fill in or do not trust the filled-in information (green) or if they have veridical foveal information under photopic viewing (cyan).

(B) Appearance task: proportion of selected stimuli reported as continuous. Discontinuous and continuous stimuli are shown in orange and red, respectively. Error bars are bootstrapped 95% CIs. Dashed lines indicate guessing without a bias. See also Figures S1 and S2.

vision alike. However, a simple heuristic is unlikely, because humans can estimate uncertainty based on their actual perceptual performance instead of using simple cues, such as contrast or eccentricity in photopic vision [26]. Second, confidence might be assessed for each stimulus individually also in scotopic vision. In this case, our finding that biases in photopic and scotopic vision were similar, suggesting that confidence is assessed at a level of processing where information about the originating photoreceptor type is lost and perceptual filling-in is completed. Such a dissociation is quite likely, because rod and cone photoreceptors converge on the same pathways at the level of retinal ganglion cells [27, 28] and filling-in is preattentive [29] and takes place in visual cortex [9], while confidence in contrast seems to be represented only further downstream in parietal [30] and prefrontal cortex [31] and the striatum [32].

Several basic properties of visual processing, such as pupil size [33] or photoreceptor sensitivity [34], are directly adjusted to the light level during dark adaptation. Our results show that this is not the case for the relative weighting of foveal and peripheral information in perceptual decision making. However, other properties, such as rod-cone interactions [35] or spontaneous cortical activity [36], are controlled by a circadian rhythm rather than by light level. Since our measurements were taken during the day, it is possible that the relative weighting of foveal and peripheral information is also controlled by a circadian rhythm. In this case, the bias for foveal information should be reduced or even reversed at night but possibly in the same way for both scotopic and photopic viewing.

While there are only few and contradictory studies about filling-in of the scotopic foveal scotoma [6, 7, 10], more is known about filling-in at the blind-spot, where photoreceptors are absent because the axons of the ganglion cells exit the eye ball. Here, even complex visual patterns can be filled in from the surround [29], and humans are overconfident for this filled-in information [5]. Filling-in has also been observed for scotomata in the fovea caused by macular disease [37], and these patients need to acquire a new preferred retinal locus for fixation [38]. Our finding of a general preference for foveal information, irrespective of whether it is veridical or not, suggests that preferences in perceptual decision making might not necessarily shift to the preferred retinal locus in those patients, leading to suboptimal perceptual decisions.

STAR★METHODS

Detailed methods are provided in the online version of this paper and include the following:

- KEY RESOURCES TABLE
- CONTACT FOR REAGENT AND RESOURCE SHARING
- EXPERIMENTAL MODEL AND SUBJECT DETAILS
- METHOD DETAILS
  - Equipment
  - Stimuli
  - Procedure
  - Design
- QUANTIFICATION AND STATISTICAL ANALYSIS
  - Analysis
  - Exclusion of data
  - Predictions
- DATA AND SOFTWARE AVAILABILITY

SUPPLEMENTAL INFORMATION

Supplemental Information can be found with this article online at https://doi.org/10.1016/j.cub.2019.02.023.

CellPress

ACKNOWLEDGMENTS

This work was supported by the European Research Council (ERC) under the European Union's Horizon 2020 Research and Innovation Program (grant agreement no. 676786) and the Deutsche Forschungsgemeinschaft (DFG, German Research Foundation) project number 222641018 – SFB/TRR 135 TP B2. We are grateful to Pascal Mamassian and Karl Gegenfurtner for comments on an earlier version of the manuscript, Christian Wolf for discussion, and Marie Carmine for helping with data collection.

AUTHOR CONTRIBUTIONS

A.H.G. and A.C.S. designed the study. A.H.G. collected data. A.H.G. and A.C.S. analyzed data. A.H.G. and A.C.S. wrote the paper.

DECLARATION OF INTEREST

The authors declare no competing interests.

Received: November 16, 2018
Revised: January 15, 2019
Accepted: February 6, 2019
Published: March 21, 2019

REFERENCES

1. Curcio, C.A., Sloan, K.R., Kalina, R.E., and Hendrickson, A.E. (1990). Human photoreceptor topography. J. Comp. Neurol. *292*, 497–523.
2. Jacobs, R.A. (2002). What determines visual cue reliability? Trends Cogn. Sci. *6*, 345–350.
3. Wässle, H., Grünert, U., Chun, M.H., and Boycott, B.B. (1995). The rod pathway of the macaque monkey retina: identification of AII-amacrine cells with antibodies against calretinin. J. Comp. Neurol. *361*, 537–551.
4. Barton, B., and Brewer, A.A. (2015). fMRI of the rod scotoma elucidates cortical rod pathways and implications for lesion measurements. Proc. Natl. Acad. Sci. USA *112*, 5201–5206.
5. Ehinger, B.V., Häusser, K., Ossandón, J.P., and König, P. (2017). Humans treat unreliable filled-in percepts as more real than veridical ones. eLife *6*.
6. Hubel, D.H. (1997). Vision in dim light. Nature *388*, 32–33.
7. He, S., and Davis, W.L. (2001). Filling-in at the natural blind spot contributes to binocular rivalry. Vision Res. *41*, 835–840.
8. Pessoa, L., Thompson, E., and Noë, A. (1998). Finding out about filling-in: a guide to perceptual completion for visual science and the philosophy of perception. Behav. Brain Sci. *21*, 781–796.
9. Komatsu, H. (2006). The neural mechanisms of perceptual filling-in. Nat. Rev. Neurosci. *7*, 220–231.
10. Linden, J. (1997). Perceptual Completion across the Fovea under Scotopic/Mesopic Viewing Conditions. In Computational neuroscience, J.M. Bower, ed. (New York: Plenum), pp. 717–722.
11. Mamassian, P. (2016). Visual Confidence. Ann. Rev. Vis. Sci. *2*, 459–481.
12. Barthelmé, S., and Mamassian, P. (2009). Evaluation of objective uncertainty in the visual system. PLoS Comput. Biol. *5*, e1000504.
13. Anstis, S.M. (1974). Letter: A chart demonstrating variations in acuity with retinal position. Vision Res. *14*, 589–592.
14. Hood, D.C., and Finkelstein, M.A. (1986). Sensitivity to light. In Handbook of Perception and Human Performance, K.R. Boff, L. Kaufman, and J.P. Thomas, eds. (New York: Wiley), pp. 1–66.
15. Bouma, H. (1970). Interaction effects in parafoveal letter recognition. Nature *226*, 177–178.
16. Brenner, E., Mamassian, P., and Smeets, J.B.J. (2008). If I saw it, it probably wasn't far from where I was looking. J. Vis. *8*, 7.
17. Toscani, M., Gegenfurtner, K.R., and Valsecchi, M. (2017). Foveal to peripheral extrapolation of brightness within objects. J. Vis. *17*, 14.
18. Herwig, A., and Schneider, W.X. (2014). Predicting object features across saccades: evidence from object recognition and visual search. J. Exp. Psychol. Gen. *143*, 1903–1922.
19. Valsecchi, M., and Gegenfurtner, K.R. (2016). Dynamic Re-calibration of Perceived Size in Fovea and Periphery through Predictable Size Changes. Curr. Biol. *26*, 59–63.
20. Ganmor, E., Landy, M.S., and Simoncelli, E.P. (2015). Near-optimal integration of orientation information across saccades. J. Vis. *15*, 8.
21. Wolf, C., and Schütz, A.C. (2015). Trans-saccadic integration of peripheral and foveal feature information is close to optimal. J. Vis. *15*, 1.
22. Gegenfurtner, K.R., Mayser, H., and Sharpe, L.T. (1999). Seeing movement in the dark. Nature *398*, 475–476.
23. Ennis, R., Toscani, M., and Gegenfurtner, K.R. (2017). Seeing lightness in the dark. Curr. Biol. *27*, R586–R588.
24. Paulun, V.C., Schütz, A.C., Michel, M.M., Geisler, W.S., and Gegenfurtner, K.R. (2015). Visual search under scotopic lighting conditions. Vision Res. *113* (Pt B), 155–168.
25. Najemnik, J., and Geisler, W.S. (2005). Optimal eye movement strategies in visual search. Nature *434*, 387–391.
26. Barthelmé, S., and Mamassian, P. (2010). Flexible mechanisms underlie the evaluation of visual confidence. Proc. Natl. Acad. Sci. USA *107*, 20834–20839.
27. Zele, A.J., and Cao, D. (2015). Vision under mesopic and scotopic illumination. Front. Psychol. *5*, 1594.
28. Demb, J.B., and Singer, J.H. (2012). Intrinsic properties and functional circuitry of the AII amacrine cell. Vis. Neurosci. *29*, 51–60.
29. Ramachandran, V.S. (1992). Filling in the blind spot. Nature *356*, 115.
30. Kiani, R., and Shadlen, M.N. (2009). Representation of confidence associated with a decision by neurons in the parietal cortex. Science *324*, 759–764.
31. Fleming, S.M., Weil, R.S., Nagy, Z., Dolan, R.J., and Rees, G. (2010). Relating introspective accuracy to individual differences in brain structure. Science *329*, 1541–1543.
32. Hebart, M.N., Schriever, Y., Donner, T.H., and Haynes, J.D. (2016). The Relationship between Perceptual Decision Variables and Confidence in the Human Brain. Cerebral Cortex *26*, 118–130.
33. Watson, A.B., and Yellott, J.I. (2012). A unified formula for light-adapted pupil size. J. Vis. *12*, 12.
34. Lamb, T.D., and Pugh, E.N., Jr. (2004). Dark adaptation and the retinoid cycle of vision. Prog. Retin. Eye Res. *23*, 307–380.
35. Ribelayga, C., Cao, Y., and Mangel, S.C. (2008). The circadian clock in the retina controls rod-cone coupling. Neuron *59*, 790–801.
36. Cordani, L., Tagliazucchi, E., Vetter, C., Hassemer, C., Roenneberg, T., Stehle, J.H., and Kell, C.A. (2018). Endogenous modulation of human visual cortex activity improves perception at twilight. Nat. Commun. *9*, 1274.
37. Zur, D., and Ullman, S. (2003). Filling-in of retinal scotomas. Vision Res. *43*, 971–982.
38. Crossland, M.D., Culham, L.E., Kabanarou, S.A., and Rubin, G.S. (2005). Preferred retinal locus development in patients with macular disease. Ophthalmology *112*, 1579–1585.
39. Kleiner, M., Brainard, D., Pelli, D., Ingling, A., Murray, R., and Broussard, C. (2007). What's new in Psychtoolbox-3. Perception *36*, 1–16.
40. Cornelissen, F.W., Peters, E.M., and Palmer, J. (2002). The Eyelink Toolbox: eye tracking with MATLAB and the Psychophysics Toolbox. Behav. Res. Methods Instrum. Comput. *34*, 613–617.

STAR★METHODS

KEY RESOURCES TABLE

REAGENT or RESOURCE	SOURCE	IDENTIFIER
Software and Algorithms		
MATLAB R2016b, including Statistics and Machine Learning Toolbox	MathWorks	https://www.mathworks.com/products/matlab.html, RRID:SCR_001622
Psychtoolbox 3, including Eyelinktoolbox	[39, 40]	http://psychtoolbox.org/, RRID:SCR_002881
Other		
Eyelink 1000+ eye tracker	SR Research	https://www.sr-research.com/products/eyelink-1000-plus/, RRID:SCR_009602
VIEWPixx monitor	VPixx Technologies	http://vpixx.com/products/viewpixx/, RRID:SCR_013271

CONTACT FOR REAGENT AND RESOURCE SHARING

Further information and requests for resources and reagents should be directed to and will be fulfilled by the Lead Contact, Alexander C. Schütz (a.schuetz@uni-marburg.de).

EXPERIMENTAL MODEL AND SUBJECT DETAILS

Overall, forty-nine observers completed one of the experiments. Twenty-five observers (twenty women, mean age = 24.1 years, range = 19-32) completed Experiment 1, thirteen of them were tested in the continuity response condition and twelve in the discontinuity response condition. Another group of eleven observers (nine women, mean age = 23.8 years, range = 19-34) completed Experiment 2. Finally, an additional group of thirteen observers (six women, mean age = 23.8 years, range = 18-44) completed the fixation manipulation experiment (Experiment 3). After exclusion, however, only forty observers with valid datasets remained. Twenty-two observers in Experiment 1 (eleven observers for each response condition), nine observers in Experiment 2, and nine observers in Experiment 3. More details about the exclusion criteria are given below.

Observers were students of Marburg University and were reimbursed for participation with 8€ per hour or partial course credit. Experiments were in accordance with the principles of the Declaration of Helsinki and ethics approval was obtained from the local ethics commission of the Department of Psychology of Marburg University (proposal number 2015-35k). All observers gave informed consent and had normal or corrected to normal vision.

METHOD DETAILS

Equipment

Stimuli were displayed using the Psychophysics Toolbox 3 [39] in MATLAB (Mathworks, Natick, MA, USA) on a VIEWPixx monitor (VPixx Technologies, Saint-Bruno, QC Canada) at a 1920 × 1080 pixel resolution and a 120-Hz refresh rate. The monitor had a size of 51.5 × 29 cm and was viewed at a distance of 61.5 cm, resulting in 40 pixels/°. The luminance output of the monitor was linearized.

Viewing conditions were manipulated in two separate experimental sessions. Measurements were performed in both scotopic and photopic range of luminance using neutral density (ND) filters (LEE Filters, Burbank, CA). The ND filters were sandwiched between polymethyl methacrylate (PMMA) layers and these arrangements were mounted in a black metal frame in front the monitor. For scotopic measurements, we used a set of ND filters to reduce the measured luminance of black, gray and white pixels from 0.10700, 52.25000, 99.02000 cd/m^2 to 0.013x10^{-4}, 6.174x10^{-4}, 11.701x10^{-4} cd/m^2 (nominal values, below the measurement threshold of a UDT Instruments Optometer 370), respectively. With these values our stimuli did not exceed the absolute cone excitation threshold of about 10x10^{-4} cd/m^2 [27] and our perceptual measurements showed that observers were not able to discriminate our stimuli inside the scotopic foveal scotoma (Figure 2A). For photopic measurements, we used two PMMA layers without any ND filter sandwiched reducing the measured luminance of black, gray and white pixels to 0.09450, 45.00000, 85.92000 cd/m^2, respectively.

Eye movements of the right eye were recorded using the Eyelink Toolbox [40] and an EyeLink 1000 (SR Research Ltd., Ontario, Canada) with a sampling rate of 1000 Hz. Participant responses were recorded via a standard keyboard.

Stimuli

Stimuli were circles with independently striped center and surround, either vertical or horizontal (Figure 1A). The diameter of the center was 0.7°, a size smaller than the central region of the fovea without rods (≅1°) [1], while the exterior diameter of the surround was 6°. The stripes pattern was a sinusoidal waveform with a spatial frequency of 1.4 cpd. This value was selected to guarantee one complete cycle inside the center. The contrast of center and surround was 0.99. The phase of the sinewave was fixed at a value of 0. The exterior edge of the surround was smoothed over 0.25°, whereas the border between the center and the surround was sharp. Center and surround were presented simultaneously and the stripes could be either continuous (C; same orientation of center and surround) or discontinuous (D; orthogonal orientation of center and surround).

Procedure

In Experiment 1, the duration of each session was around 80 min, divided in 20 min of adaptation to the background luminance, 10 min for demonstration and eye tracking calibration, and 40-50 min of data collection. After a third of trials were completed (33%, 66%) observers received visual feedback with information about the number of trials, and performed a short eye movement task in which they had to follow a fixation cross appearing at different locations on the screen. This task was designed to force observers to move their eyes and to avoid effects related to long fixation periods at the same location.

In each trial, a fixation cross was presented at the screen center and observers were instructed to press the space-bar to begin the trial (Figure 1B). Afterward, the fixation cross was replaced by two aligned vertical lines with a gap of 7° and observers had to continue fixating at the center of the gap for the remainder of the trial. After a random time between 1 and 1.5 s, a stimulus was presented for 300 ms on the horizontal meridian at 0° (screen center), or at 4° or 8° left or right from the center. After 700 ms, the second stimulus was presented in the same or in another location (but always on the same side of the screen). Half of the observers were instructed to indicate which stimulus in the sequence was the continuous one (continuity response). The other half of the observers were instructed to indicate which stimulus in the sequence was the discontinuous one (discontinuity response). They were shown an example of a continuous and a discontinuous stimulus before the experiment and they completed several demonstration trials before the data collection started, 10 during the first session and 5 during the second one. If they mentioned during the demonstration that both stimuli are very similar, we instructed them to choose the one that for them was the best example of a continuous (or discontinuous) stimulus. After a trial, observers received auditory feedback when they moved their eyes more than 2° from the screen center and these trials were flagged for future exclusion during the offline analysis.

In the same-eccentricity conditions, both stimuli in a trial were presented at the same location (0°, 4° or 8°). In the different-eccentricity conditions, they were presented at different eccentricities (0°|4°, 0°|8° or 4°|8°). In both cases, both stimuli in a trial were presented on the same side of the screen, either on the left or on the right side (in a counterbalanced way).

Experiment 2 was similar to Experiment 1, but now observers first needed to select which stimulus in the sequence they want to judge (selection task), and then report if the selected stimulus appeared continuous or discontinuous to them (appearance task). Observers completed several demonstration trials before the data collection started, 15 during the first session and 8 during the second one. To reduce the duration of the sessions, we only tested different-eccentricity conditions.

Experiment 3 was also similar to Experiment 1, except that the fixation at the beginning of each trial was at the screen center, or 4° left or right from screen center (Figure S3). The initial fixation position was randomized and counterbalanced across the session. Given that Experiment 3 required more trials, we performed this experiment only under the photopic viewing and without the previous background luminance adaptation. In addition, we tested only the different-eccentricity conditions.

Design

In Experiment 1, we tested 24 interleaved conditions (Table S1). These 24 conditions (3 eccentricities (0°, 4°, 8°) x 2 temporal sequences (CD or DC) in the same-eccentricity conditions, and 3 eccentricities (0°|4°, 0°|8°, 4°|8°) x 3 stimulus conditions (CC, CD or DC) x 2 temporal sequences in the different-eccentricity conditions) were measured 12 times (3 repetitions x 2 locations on the screen (left, right) x 2 orientations (vertical, horizontal)). This resulted in 288 trials.

To measure discrimination performance in same-eccentricity conditions, we used 6 different conditions in which both stimuli were presented at the same eccentricity (0°, 4° or 8°). For each eccentricity there were two different possibilities, first stimulus continuous and second stimulus discontinuous (CD), and vice-versa (DC).

To measure perceptual biases in different-eccentricity conditions, we used 18 different conditions in which the first and the second stimuli were presented at different eccentricities (0°|4°, 0°|8°, 4°|8°). In addition to the CD and DC possibilities, there was an ambiguous condition in which both first and second stimuli were continuous (CC). In nine of these 18 conditions, stimulus presentations followed a sequence of first-second stimulus locations opposite to the others nine (i.e., CD at 0°|4°, CD at 4°|0°).

Responses for conditions with the same eccentricities but reversed sequences – for example 0°|4° and 4°|0° – were normalized to the lower eccentricity first case (0°|4°) and grouped together. In addition, DC and CD values for the same-eccentricity conditions (first and second stimulus at the same eccentricity) were considered together for each eccentricity.

In Experiment 2, the same 18 different-eccentricity conditions from Experiment 1 were tested interleaved. These 18 conditions (3 eccentricities (0°|4°, 0°|8°, 4°|8°) x 3 stimulus conditions (CC, CD or DC) x 2 temporal sequences), were measured 16 times (4 repetitions x 2 locations on the screen (left, right) x 2 orientations (vertical, horizontal)). This resulted in 288 trials.

In Experiment 3, we tested 36 interleaved conditions. These 36 conditions (3 initial fixation positions (−4°, 0°, 4°) x 2 stimulus eccentricities (0°|4°, 0°|−4°) x 3 stimulus conditions (CC, CD, DC) x 2 temporal sequences, were measured 12 times (6 repetitions x 2

orientations (vertical, horizontal)). This resulted in 432 trials. The retinal eccentricities were the same as in the condition 0°|4° (or −4°|0°) in Experiment 1 but using different screen locations now. For example, with the fixation at 4° from the screen center, the eccentric locations on the screen were either left (0°) or right (8°). Responses for stimuli conditions with opposite order – for example −8°|−4° and −4°|−8° – were normalized to the lower eccentricity first case (−4°|−8°) and grouped together.

QUANTIFICATION AND STATISTICAL ANALYSIS

Analysis

In the same-eccentricity conditions of Experiment 1, the proportion of correct responses was calculated and analyzed. In the different-eccentricity conditions of Experiment 1, the proportion of less-eccentric stimuli reported as continuous was calculated. Since there were no differences between observers in the continuity or discontinuity response condition, both groups were merged and analyzed together. In Experiment 2, we calculated the proportion of less-eccentric stimuli selected in the selection task. For the appearance task, we calculated the proportion of selected stimuli reported as continuous. In Experiment 3, the proportion of the fixated stimulus reported as continuous was calculated (Figure S3).

We report means and 95% confidence intervals in the results section and Figures 2 and 3. Confidence intervals were bootstrapped, using 20000 bootstrap samples and a bias corrected and accelerated percentile method.

To analyze the additivity of biases in the comparison task of Experiment 1 and the selection task of Experiment 2, we summed values of the 0°|4° and the 4°|8° conditions and compared them to the 0°|8° condition (Figure S1). Before summation, proportion data were linearized using a logit transformation:

$$y = \log\left(\frac{y}{(1-y)}\right) \tag{1}$$

To be able to transform proportion data of 0 or 1, we added or subtracted a constant of 48^{-1}, representing half of the maximum resolution that we could achieve with 24 repetitions in each condition.

To analyze potential changes in biases over the course of the experiment, we ordered trials according to their number within each condition and averaged across observers for each trial number. Linear trends were assessed using a regression analysis (Figure S2).

Exclusion of data

Individual trials were considered invalid if the observer's fixation deviated more than 2° of visual angle from the point indicated by the initial fixation cross within a time window of 1320 ms in which the test stimuli were presented. In Experiment 1, data from observers were excluded from further analysis if the proportion of valid trials in any of the grouped conditions was less than 50% (twelve trials). In Experiment 2, to be consistent despite there were more repetitions per condition, the required minimum number of valid trials in the grouped conditions was also twelve trials. In Experiment 3, however, the conditions were not grouped for the analysis, and given that the number of repetitions per condition was twelve, data from observers were excluded if the number of valid trials was less than 50% (six trials) in any condition. Accordingto these criteria, we excluded one observer in Experiment 1 (continuity response condition) and three observers in Experiment 3.

To make sure that observers performed the perceptual tasks properly, we excluded observers when their performance in non-ambiguous conditions was too low. In Experiment 1, observers with a perceptual accuracy less or equal to 75% correct in any unambiguous same-eccentricity condition (except for 0° eccentricity under scotopic conditions), were excluded. This applied to two observers (one of them in the discontinuity response condition and the other one in the continuity response condition). In Experiment 3, one observer with a perceptual accuracy less or equal to 75% was excluded. In Experiment 2, two observers with a perceptual accuracy less or equal to 75% in any condition of the appearance task (except for a discontinuous stimulus presented at 0° eccentricity under scotopic conditions) were excluded. Since the individual bias of observers in the selection task determined in how many trials they reported the appearance of a given stimulus and eccentricity condition, we included only those conditions for the appearance task that contained at least 14 trials.

The results of all experiments were qualitatively unaffected by the specified exclusions of trials and observers.

Predictions

We predicted decisions of unbiased observers in the comparison task of Experiment 1 and the selection task of Experiment 2 for two scenarios under scotopic viewing: if observers experience a perceptual gap at the scotopic foveal scotoma or experience filling-in but do not trust this inferred information (perceptual-gap) and if observers experience filling-in and trust this inferred information as much as veridical information from the periphery (filling-in). In addition, we predicted decisions of unbiased observers under photopic viewing, when all eccentricities are weighted equally.

In Experiment 1 (Figure 2B), according to the perceptual-gap prediction, observers should base their decisions under scotopic viewing on peripheral stimuli and ignore foveal stimuli, because they are aware that they do not have veridical information about foveal stimuli. Therefore, independently of which stimulus is shown at the fovea, it should always be chosen as continuous when the peripheral stimulus is discontinuous and never chosen as continuous when the peripheral stimulus is continuous. According to the filling-in prediction, observers perceive any foveal stimulus as continuous and weight foveal and peripheral information equally for their decisions under scotopic viewing. Therefore, independently of which stimulus is shown at the fovea, it should always be

chosen as continuous when the peripheral stimulus is discontinuous. When the peripheral stimulus is continuous, the foveal and the peripheral stimulus will both be perceived as continuous and therefore should be chosen as continuous equally often. This should also be the case when both stimuli are continuous. Under photopic viewing, observers should report the correct stimulus as continuous when a continuous and a discontinuous stimulus are shown and report both stimuli equally often when two continuous stimuli are shown.

In Experiment 2 (Figure 3A), according to the perceptual-gap prediction, observers should never select foveal stimuli for the subsequent appearance task, because they are aware that they cannot judge these stimuli veridically. According to the filling-in prediction, observers should not exhibit any preference in the selection task and select foveal and peripheral stimuli equally often in all conditions. This should also be the case under photopic viewing.

DATA AND SOFTWARE AVAILABILITY

Raw data are available at https://doi.org/10.5281/zenodo.2358129.

Current Biology, Volume *29*

Supplemental Information

Humans Trust Central Vision More

Than Peripheral Vision Even in the Dark

Alejandro H. Gloriani and Alexander C. Schütz

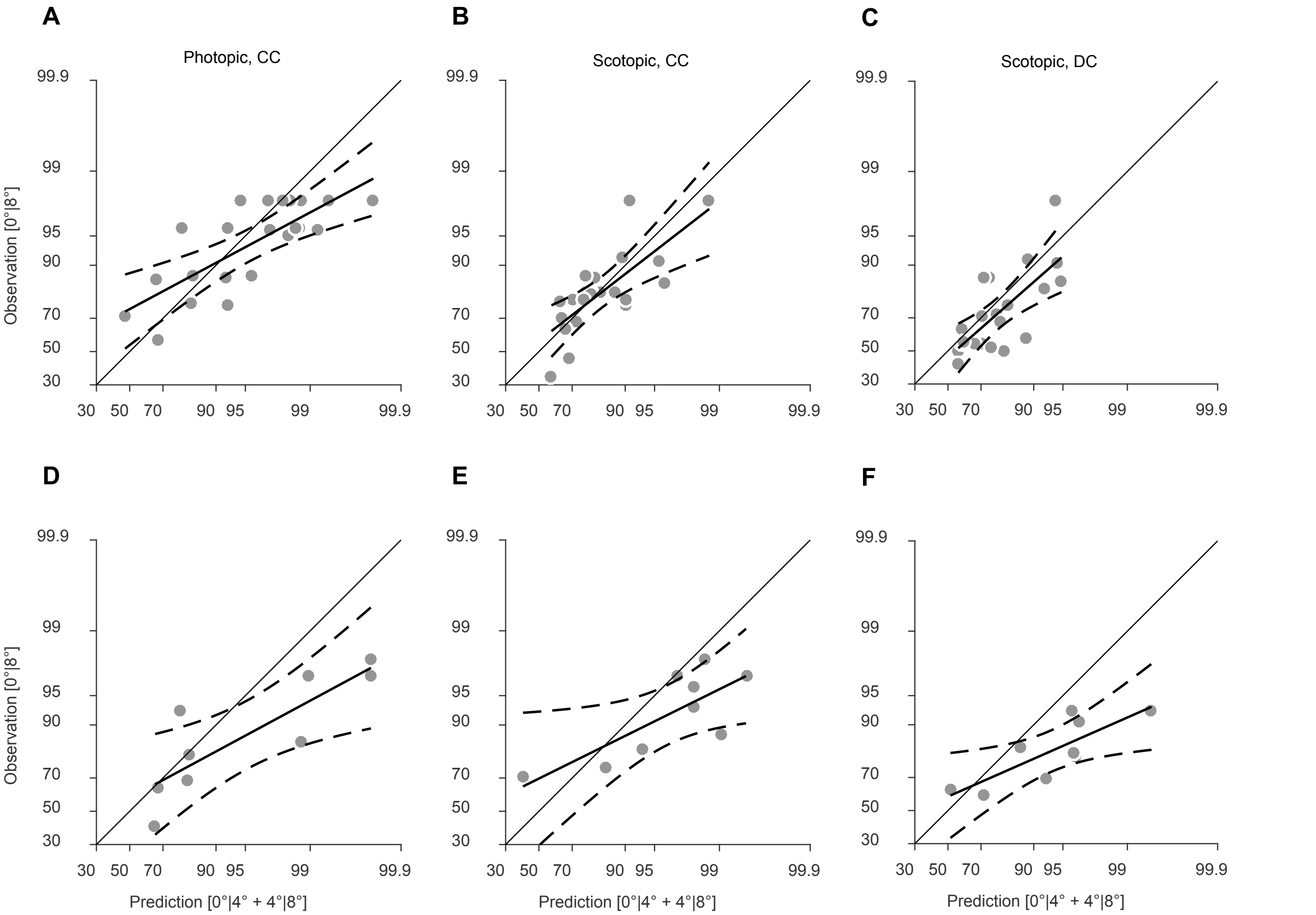

Figure S1. Additivity of biases at different eccentricities in the comparison task of Experiment 1 and the selection task of Experiment 2 (related to Figures 2 and 3)

The sum of the biases for 0°|4° and 4°|8° is shown on the x-axis and the bias for 0°|8° is shown on the y-axis. Before summation, a logit transformation was applied to the proportion data (Equation 1). Gray symbols indicate individual observers; the solid line indicates a linear regression and dashed lines indicate 95% confidence intervals of the regression. All regressions were significant.

(A) Experiment 1, photopic viewing, two continuous stimuli ($b = 0.54$, $R^2 = 0.59$, $F(1,20) = 28.21$, $p < 0.001$).

(B) Experiment 1, scotopic viewing, two continuous stimuli ($b = 0.77$, $R^2 = 0.56$, $F(1,20) = 24.98$, $p < 0.001$)

(C) Experiment 1, scotopic viewing, discontinuous and continuous stimuli at the lower and higher eccentricity, respectively ($b = 0.88$, $R^2 = 0.54$, $F(1,20) = 23.52$, $p < 0.001$). Since the DC condition at 4°|8° was not ambiguous, we used the CC condition at 4°|8° for this prediction.

(D) Experiment 2, selection task, photopic viewing, two continuous stimuli ($b = 0.54$, $R^2 = 0.71$, $F(1,7) = 17.02$, $p = 0.004$).

(E) Experiment 2, selection task, scotopic viewing, two continuous stimuli ($b = 0.49$, $R^2 = 0.60$, $F(1,7) = 10.69$, $p = 0.014$).

(F) Experiment 2, selection task, scotopic viewing, discontinuous and continuous stimuli at the lower and higher eccentricity, respectively ($b = 0.44$, $R^2 = 0.66$, $F(1,7) = 13.35$, $p = 0.008$). Since the DC condition at 4°|8° was not ambiguous, we used the CC condition at 4°|8° for this prediction.

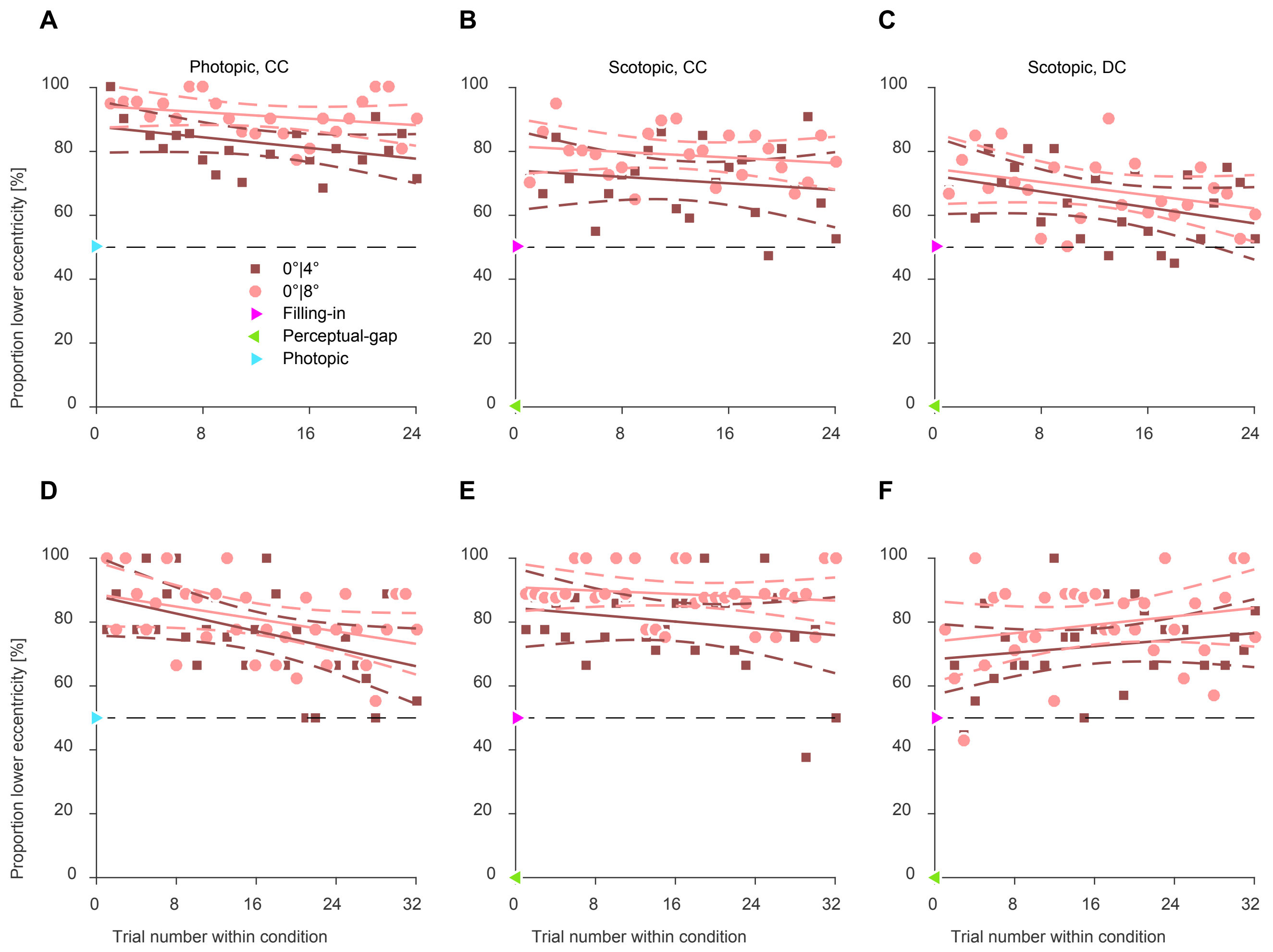

Figure S2. Distribution of biases across trials in the comparison task of Experiment 1 and the selection task of Experiment 2 (related to Figures 2 and 3)

Circles and squares show averages across observers for each trial within a condition. The solid line indicates a linear regression and dashed lines indicate 95% confidence intervals of the regression. 0°|4° and 0°|8° conditions are shown in dark and bright red, respectively. Triangles indicate predictions (STAR Methods) if observers trust the filled-in information at the fovea (magenta) or either do not fill-in or do not trust the filled-in information (green) or if they have veridical foveal information under photopic viewing (cyan).

(A) Experiment 1, photopic viewing, two continuous stimuli (0°|4°: $b = -0.42$, $R^2 = 0.14$, $F(1,22) = 3.66$, $p = 0.069$; 0°|8°: $b = -0.25$, $R^2 = 0.08$, $F(1,22) = 1.86$, $p = 0.186$).

(B) Experiment 1, scotopic viewing, two continuous stimuli (0°|4°: $b = -0.25$, $R^2 = 0.03$, $F(1,22) = 0.57$, $p = 0.459$; 0°|8°: $b = -0.22$, $R^2 = 0.04$, $F(1,22) = 0.87$, $p = 0.361$)

(C) Experiment 1, scotopic viewing, discontinuous and continuous stimuli at the lower and higher eccentricity, respectively (0°|4°: $b = -0.62$, $R^2 = 0.14$, $F(1,22) = 3.72$, $p = 0.067$; 0°|8°: $b = -0.52$, $R^2 = 0.12$, $F(1,22) = 3.02$, $p = 0.096$).

(D) Experiment 2, selection task, photopic viewing, two continuous stimuli (0°|4°: $b = -0.69$, $R^2 = 0.20$, $F(1,30) = 7.27$, $p = 0.011$; 0°|8°: $b = -0.49$, $R^2 = 0.15$, $F(1,30) = 5.50$, $p = 0.026$).

(E) Experiment 2, selection task, scotopic viewing, two continuous stimuli (0°|4°: $b = -0.27$, $R^2 = 0.03$, $F(1,30) = 1.08$, $p = 0.308$; 0°|8°: $b = -0.13$, $R^2 = 0.02$, $F(1,30) = 0.70$, $p = 0.411$).

(F) Experiment 2, selection task, scotopic viewing, discontinuous and continuous stimuli at the lower and higher eccentricity, respectively (0°|4°: $b = 0.25$, $R^2 = 0.04$, $F(1,30) = 1.24$, $p = 0.274$; 0°|8°: $b = 0.33$, $R^2 = 0.05$, $F(1,30) = 1.59$, $p = 0.217$).

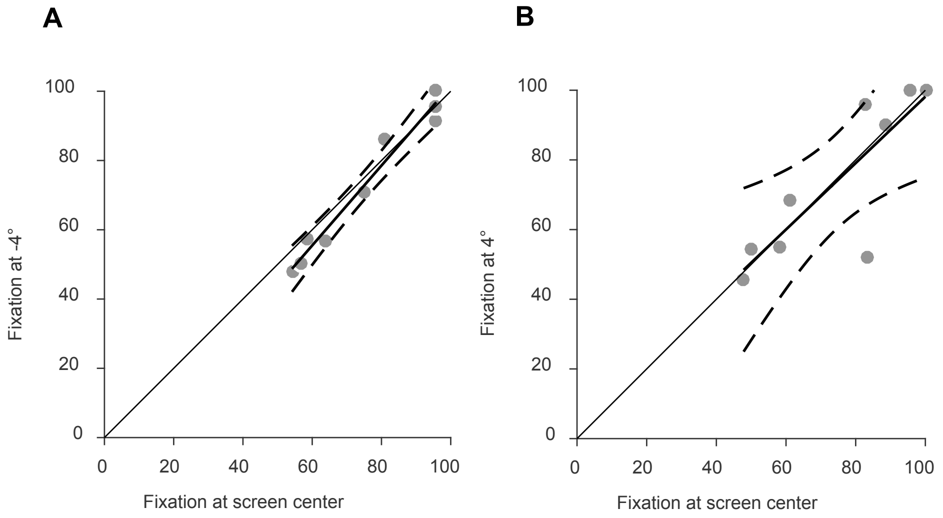

Figure S3. Influence of retinal and screen location on perceptual biases in Experiment 3 (n=9) (related to STAR Methods)

To study if perceptual biases depended on the location on the screen or on the retina, we varied the fixation location in three conditions (-4°, 0° and 4°) under photopic viewing. In each trial two continuous stimuli were presented at the fixation location and either 4° left or right from fixation. This way we could compare two conditions with matching stimulus locations at the screen, but one with fixation at the screen center and one with fixation 4° left or right from the screen center. For each condition we calculated the proportion of the fixated stimulus reported as more continuous. If biases depend on retinal location, there should be a positive relationship between central and eccentric fixation conditions. If biases depend on screen location, there should be a negative relationship between central and eccentric fixation conditions. Gray symbols indicate individual observers; the solid line indicates a linear regression and dashed lines indicate 95% confidence intervals of the regression. (A) Fixation at screen center vs. fixation at -4° eccentricity ($b = 1.15$, $R^2 = 0.97$, $F(1,7) = 203.68$, $p < 0.001$). (B) Fixation at screen center vs. fixation at 4° eccentricity ($b = 0.95$, $R^2 = 0.70$, $F(1,7) = 16.40$, $p = 0.005$).

Condition		1	2	3	4	5	6	7	8	9	10	11	12	13	14	11	12	17	18	19	20	21	22	23	24
Location		Same eccentricity						Different eccentricity																	
Stimulus								CD		CD		CD		DC		DC		DC		CC		CC		CC	
1st	Ecc	0		4		8		0	4	0	8	4	8	0	4	0	8	4	8	0	4	0	8	4	8
	Rot	90	0	90	0	90	0	0	90	0	90	0	90	90	0	90	0	90	0	0	0	0	0	0	0
2nd	Ecc	0		4		8		4	0	8	0	8	4	4	0	8	0	8	4	4	0	8	0	8	4
	Rot	0	90	0	90	0	90	90	0	90	0	90	0	0	90	0	90	0	90	0	0	0	0	0	0

Table S1. Conditions in Experiments 1 and 2 (related to Figure 1 and STAR Methods)

Condition: The temporal order of the dark shaded conditions was reversed when averaging out sequence effects. Location: Same eccentricity conditions were only tested in Experiment 1. Stimulus: C indicates continuous, D indicates discontinuous; light shaded labels indicate ambiguous conditions (both stimuli perceived as continuous) in scotopic and photopic viewing; dark shaded labels indicate ambiguous conditions (both stimuli perceived as continuous) only in scotopic viewing. Ecc.: Eccentricity of stimulus in °. Rot.: Difference between center and surround orientation in °.
